# Supplementary figures and images for: A single intra-articular dose of vitamin D analog calcipotriol alleviates synovitis without adverse effects in rats
Source: PLoS One. 2021 Apr 20;16(4):e0250352. doi: 10.1371/journal.pone.0250352 (PMC8057567; doi:10.1371/journal.pone.0250352)

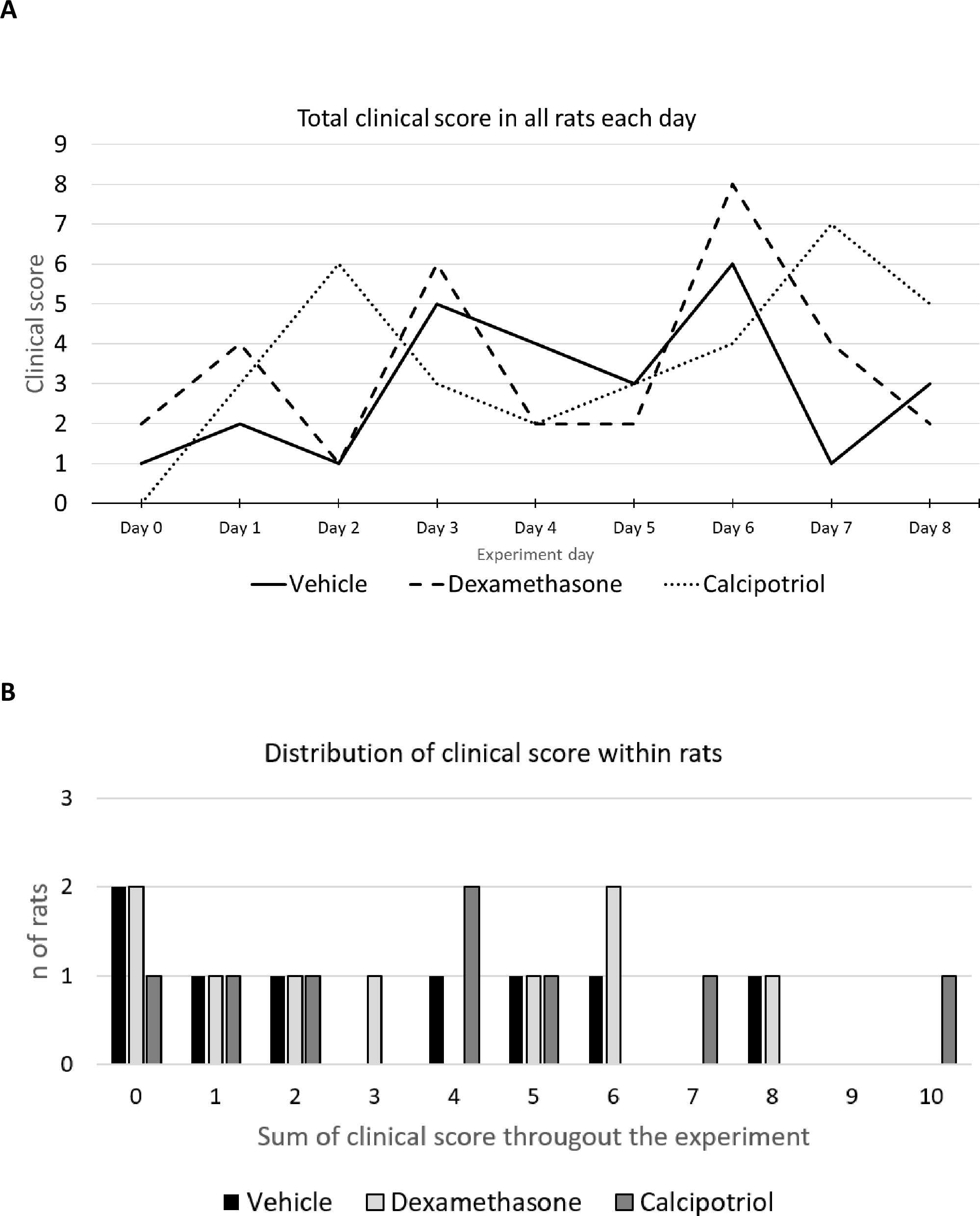

Supplement: S1 Fig — A. The sum of clinical scores on each day during the experiment in different arthritis groups. Comparing the nine-day-sum of all scores, there was no significant difference between the arthritis groups (p = 0.881 in Kruskal-Wallis test). B. The amount of rats according to the nine-day sum score at the end of the experiment. All of the rats had relatively low scores and showed practically no pain behaviour or difficulties in moving. The scores were given daily to each arthritic rat using the scoring sheet in S1 Table. The average scores were minimal, in average <1 every day in every group and thus, the scoring is represented as sums. (TIF) [file pone.0250352.s001.tif]

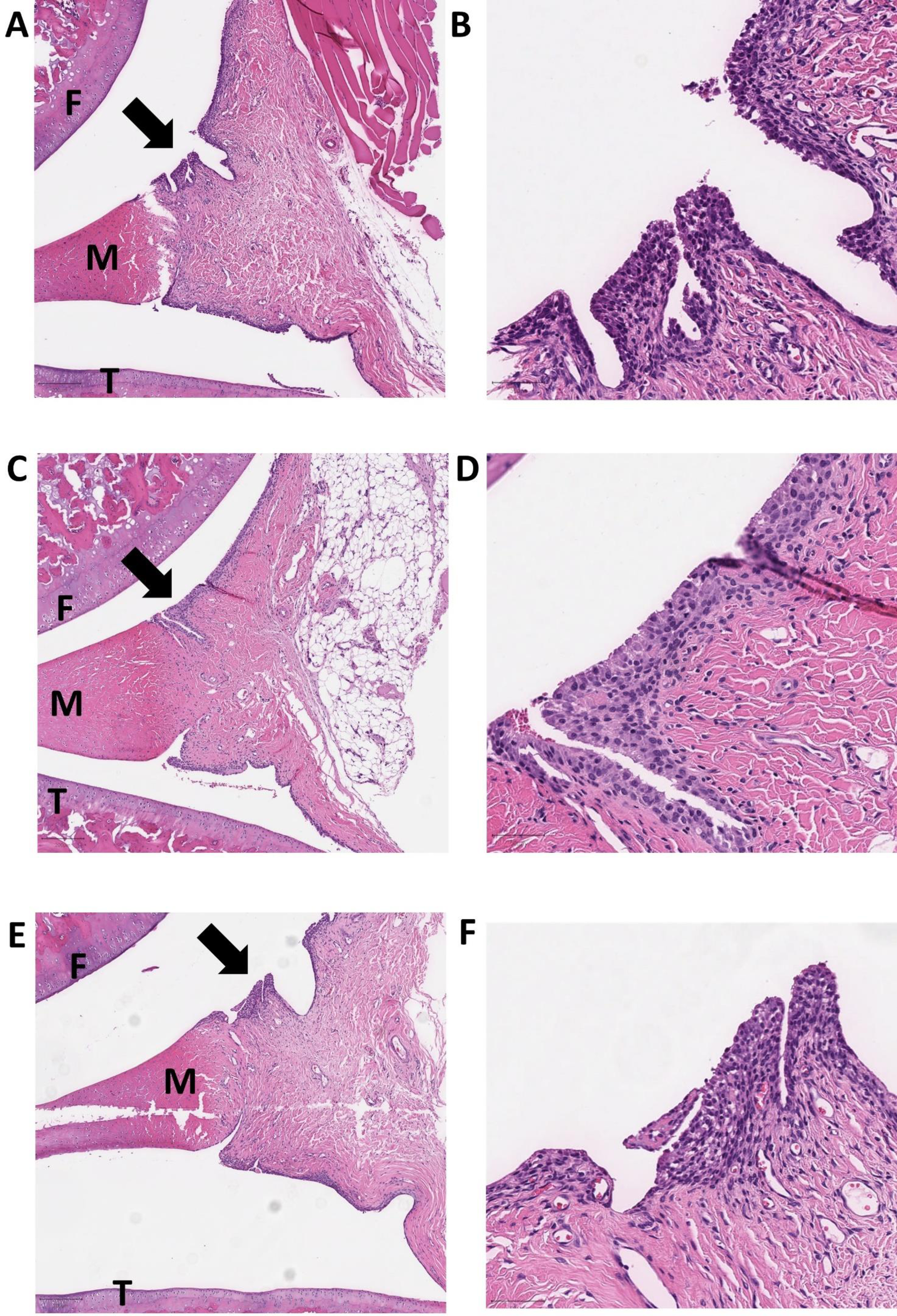

Supplement: S2 Fig — From each group, the highest grade was chosen. All images are from posterior recesses with HE staining; 5x magnification on the left, 20x magnification on the right panel. In pictures A and B, a strong synovitis in a vehicle-treated rat is seen. In C-D, a slightly milder synovitis is seen in a calcipotriol-treated rat. Pictures E-F represent a dexamethasone-treated rat with strong synovitis. Also note the thinner synovium in the tibial side of calcipotriol-treated knee (C) compared to other knees (A, E). (TIF) [file pone.0250352.s002.tif]
