# Supplementary material for: A single intra-articular dose of vitamin D analog calcipotriol alleviates synovitis without adverse effects in rats
Source: PLoS One. 2021 Apr 20;16(4):e0250352. doi: 10.1371/journal.pone.0250352 (PMC8057567; doi:10.1371/journal.pone.0250352)
Supplement: S1 Table — The scoring sheet was used for clinical scoring on a daily basis for each arthritic rat. The results are shown in S2 Fig. (DOCX) [file pone.0250352.s003.docx]

| ID of the rat: | Initial body weight: | |  |  |  |  | Date/clock | |  |  |  |  |
| --- | --- | --- | --- | --- | --- | --- | --- | --- | --- | --- | --- | --- |
|  | Humane endpoint limit (80 %): | |  |  |  |  |  | |  |  |  |  |
| Body weight | | |  |  |  |  |  | |  |  |  |  |
| Change from the initial body weight (g) | | |  |  |  |  |  | |  |  |  |  |
| Use of hind legs | Right / left leg | | R | L | R | L | R | L | R | L | R | L |
|  | Normal | 0 |  |  |  |  |  |  |  |  |  |  |
|  | Slight limp | 1 |  |  |  |  |  |  |  |  |  |  |
|  | Substantial limp | 2 |  |  |  |  |  |  |  |  |  |  |
|  | Does not bear weight at all | 3 |  |  |  |  |  |  |  |  |  |  |
| Pain response of the knee/knees to a light squeeze | No response | 0 |  |  |  |  |  |  |  |  |  |  |
|  | Slight response | 1 |  |  |  |  |  |  |  |  |  |  |
|  | Moderate response | 2 |  |  |  |  |  |  |  |  |  |  |
|  | Strong response | 3 |  |  |  |  |  |  |  |  |  |  |
| Activity in the cage | Normal | 0 |  |  |  |  |  | |  |  |  |  |
|  | Moves slower/less than normal | 1 |  |  |  |  |  | |  |  |  |  |
|  | Moves when touched but not spontaneously | 2 |  |  |  |  |  | |  |  |  |  |
|  | No movement when touched | 3 |  |  |  |  |  | |  |  |  |  |
| Fur | Shiny and smooth | 0 |  |  |  |  |  | |  |  |  |  |
|  | Unkempt, dull | 1 |  |  |  |  |  | |  |  |  |  |
|  | Moderately ruffled | 2 |  |  |  |  |  | |  |  |  |  |
|  | Very ruffled / piloerection | 3 |  |  |  |  |  | |  |  |  |  |
| Facial expression | Normal, eyes fully open | 0 |  |  |  |  |  | |  |  |  |  |
|  | Eyes half-open, mild to moderate pain face | 1 |  |  |  |  |  | |  |  |  |  |
|  | Eyes strongly squinted or closed, severe pain face | 2 |  |  |  |  |  | |  |  |  |  |
| Vocalization | No vocalization | 0 |  |  |  |  |  | |  |  |  |  |
|  | Vocalizes on touching the knee | 1 |  |  |  |  |  | |  |  |  |  |
|  | Vocalizes spontaneously in the cage | 2 |  |  |  |  |  | |  |  |  |  |
| Tooth grinding | Not at all | 0 |  |  |  |  |  | |  |  |  |  |
|  | A little / Occasionally | 1 |  |  |  |  |  | |  |  |  |  |
|  | Moderately / A lot | 2 |  |  |  |  |  | |  |  |  |  |

Points in total 0-24
