# Supplementary material for: A single intra-articular dose of vitamin D analog calcipotriol alleviates synovitis without adverse effects in rats
Source: PLoS One. 2021 Apr 20;16(4):e0250352. doi: 10.1371/journal.pone.0250352 (PMC8057567; doi:10.1371/journal.pone.0250352)
Supplement: Attachment — Submitted filename: Response to Reviewers.docx [file pone.0250352.s004.docx]

**RESPONSE TO REVIEWERS COMMENTS**

We thank you for giving us the opportunity to submit a revised draft of my manuscript titled “**A single intra-articular dose of vitamin D analog calcipotriol alleviates synovitis without adverse effects in rats**” in Plos One. We are grateful to the reviewers for their insightful comments on our paper. We used “track changes” to see the modifications in original paper.

Here is a point-by-point response to the reviewers’ comments and concerns.

Reviewer #1: Dear Editor,
Thank you for inviting me as a reviewer. Following the review of the manuscript, entitled, “A single intra-articular dose of vitamin D analog calcipotriol alleviates synovitis without adverse effects in rats” by Johanna Huhtakangas, Jere Huovinen, Sakari Laaksonen, Hanna-Marja Voipio, Olli Vuolteenaho, Mikko A.J. Finnilä, Jerome Thevenot, Petri P. Lehenkari, I recommend that it should be revised taking into given below suggestions.

1. In the Introduction, line 33-35, “In accordance with this, prophylactic treatment with vitamin D molecules have also shown disease modifying effects in some surgical models of OA in rodents in the onset of OA (10-12) but the beneficial effect was lost in the in the progression phase of OA (10-11)” are little confusing, please rephrase this.

***Author action:*** *We thank the reviewer for all comments. We modified the text and it should be clearer now.*

2. Line 35, “beneficial effect was lost in the in the progression phase of OA”, the words “in the” are redundant.

***Author action:*** *The redundancy is now omitted.*

3. In the Introduction, lines 47-48, “The aim of this study was to test hypothesis that calcipotriol that is shown to have anti-inflammatory effects on synoviocytes in vitro (4), would also alleviate arthritis in vivo in rat ZIA model” are little confusing, please rephrase this.

***Author action:*** *This sentence is rephrased now.*

4. Line 110 recheck the spelling of “chemiluminesence” and remove following an extra period.

***Author action****: This is corrected now.*

5. Line 114, the correct word will be “facial expression”.

***Author action****: This is corrected.*

6. Line 126, “and the rat were always positioned”, the proper verb should be “was” or change the subject word to “rats”.

***Author action****: The sentence is now in a plural form.*

7. Section “Preparation and analysis of the histological specimen”, line number 154, “the were cut into 5μm sagittal slices with a microtome”, clarify this sentence.

***Author* action**: “*Sections” was added as a substantive.*

8. Line 175, “175 temperature and knee calibers between days 0 and 1 was studied”, replace the word “was” with “were”.

***Author action:*** *The suggested replacement is done now.*

9. Section “clinical characteristics”, line number 194, “The relative weight loss was greater in the dexamethasone group than in the other arthritis groups on days 2–3 (6% vs. 3-4%, respectively, p<0.05)”, elaborate the cause and consequences of this observation.

***Author action:*** *We thank for the comment. The result is now discussed further in the first paragraph of discussion as follows:*

*" Dexamethasone caused significantly more weight reduction in two days after administering the drug, probably due to decreased appetite, as the weight loss was rapidly recovered in two following days. Previously hydrocortisone has been shown to reduce food intake in SD rats among other adverse effects (24).”*

10. Line 227,” The data is expressed as mean ± SD.”, replace the “is” with “are”. Amend in the subsequent occurrence too.

***Author action****: This is corrected now.*

11. The whole section “Thermal imaging (starting from line 235)” is quite confusing. Add a few lines to explain the rationale of “The increase in average temperature 240 of the right knee from day 0 to day 1 was 0.74 °C (from 35.02 to 35.76°C, p<0.0005) and the left 241 knee 0.59°C (from 35.04 to 35.63°C, p<0.0005) (right vs. left knee p=0.011), with no significant 242 increase in the surface temperature of the abdomen (from 34.97 to 35.09°C, p=0.356).”

***Author action****: Thermal imaging section has been clarified by adding a few sentences. “The increase in average temperature of the right knee from day 0 to day 1 was 0.74 °C (from 35.02 to 35.76°C, p<0.0005) that reflects vasodilatation caused by inflammation. A smaller elevation of average temperature, 0.59°C, was seen in the saline injected left knees (from 35.04 to 35.63°C, p<0.0005)* that is *caused by the injection itself (on day 1 right vs. left knee difference p=0.017 in one-sample Wilcoxon signed-rank test compared against zero). No significant increase in the surface temperature of the abdomen (from 34.97 to 35.09°C, p=0.356) was detected”*

12. Line 286, “Fig 5. Histology the right knees of the arthritic and control rats on day 8”, it should be “Histology of the…….”.

***Author action****: The sentence is now corrected.*

13. Line 298, “on day 1 in arthritic rats versus controls (p=0.002), which may indicate increased consumption”, the period is missing at the end of this sentence.

***Author action****: The period is now added.*

Thanks,
Muhammad Muaaz Aslam

Reviewer #2: The manuscript provides preliminary work dedicated to study the effect of calcipotriol on synovitis using a Zymosan-induced arthritis (ZIA) rat model. The overall set of data seems well presented and the discussion section in the manuscript is well-written. However, there are a few issues that need to be resolved before the manuscript can be published –

1. The abstract needs to be revised as it neither introduces the field properly, nor it discusses the question which could be answered with the current work. Hence, it fails to provide the importance to the work. Kindly, rewrite the abstract accordingly.

***Author action****: We thank for the comment. A few sentences at the beginning and end of the abstract have been added. The abstract should summarize now better the main findings of the work.*

2. Why is the table in Supporting Information S1 empty? Kindly, provide the required data in the table.

***Author response:*** *We thank for the comment. The presented table was filled for each rat separately and is meant to clarify the methodology of the clinical scoring. The results are discussed in Figure S2.*

***Author action****: To avoid confusion, the legends of table S1 and figure S2 were rewritten as follows:*

***S1 Table. Clinical scoring sheet of the rats, English translation.*** *The scoring sheet was used for clinical scoring on a daily basis for each arthritic rat. The results are shown in S2 Fig.*

***S2 Fig. The clinical scores of the rats during the study.*** *A.* *The sum of clinical scores on each day during the experiment in different arthritis groups. Comparing the nine-day-sum of all scores, there was no significant difference between the arthritis groups (p=0.881 in Kruskal-Wallis test). B. The amount of rats according to the nine-day sum score at the end of the experiment. All of the rats had relatively low scores and showed practically no pain behaviour or difficulties in moving. The scores were given daily to each arthritic rat using the scoring sheet in S1 Table. The average scores were <1 every day in every group and thus, the scoring is represented as sums.*

3. Please, provide more details on Figure 5. The Z-planes appear to be different in the sections. Kindly, explain how the sectioning was performed in order to reach same planes for analysis. Extra images should be provided as this links to the main conclusion of the study. Quantitation of the thickness of synovial membrane may help.

***Author response:*** *We thank for the comment. The z-planes were chosen near the midline approaching from the medial side, preferentially patella not visible, and the femur completely visible in the section. This is now added in Methods and legend of Figure 5.*

*The thickness of the synovial membrane was not measured, as we decided to follow the OARSI guideline for assessing osteoarthritis in rats. The synovial thickness is directly proportional to the number of cell layers in the lining layer, which was the main criterion in grading that was used. Extra images presenting the rats with the highest scores of synovial grading in each arthritic group are shown in Supporting Information (S3).*

4. Figure 6 is too large and have excessive number of panels. To simplify, the data can be grouped based on significance levels or type of parameters and the relevant panels can be kept in Figure 6, along with proper description in respective result section. Rest of the data can go to Supporting Information. More elaboration on this huge dataset will be helpful. Most data in the figure 6, appear to be non-significant and the substantially tall error bars make the data less convincing. Kindly, provide clear description on p-values between samples. For instance, the first penal of Figure 6 indicating significant differences among the Day 1 values in IL-13 levels is not convincing. Though, compared to the control it may stand significant. In such case, it should be properly indicated to guide the readers. Also, why the vehicle would induce cytokines? Please, explain.

***Author response****: We thank for the comment. The significance of the results is hard to observe from the error bars, as the three arthritis groups were pooled in the analysis, as described in the methods (the rats had not received the drug on day 1 sampling point).*

***Author action****: To show the result more clearly, we created a new figure with all cytokines presented control vs. arthritis (dexamethasone, calcipotriol and vehicle pooled) rats as below. All data is gathered before study drug administration on day 1 and therefore cytokines are induced in the vehicle group, as well.*

Relative concentration (mean ± SD)

|  | Arthritis | Control |
| --- | --- | --- |
| IL-13 p=0.003 | 1.91554 | 1 |
| IL-10 p=0.872 | 0.849178 | 1 |
| IL-18 p=0.013 | 1.39057 | 1 |
| IL-1α p=0.017 | 1.44372 | 1 |
| IL-2 p=0.577 | 1.19267 | 1 |
| M-CSF p<0.0005 | 1.60991 | 1 |
| IL-1β p=0.843 | 0.901596 | 1 |
| IL-4 p=0.009 | 1.2163 | 1 |
| IFN-γ p<0.0005 | 1.56843 | 1 |
| MIP3a p=0.303 | 1.0845 | 1 |
| GM-CSF p=0.627 | 0.937244 | 1 |
| IL-7 p=0.602 | 0.982203 | 1 |
| TNF-α p=0.002 | 1.97846 | 1 |
| VEGF p=0.529 | 0.889718 | 1 |
| MCP-1 p=0.325 | 0.606627 | 1 |
| IL-5 p=0.113 | 1.21049 | 1 |
| G-CSF p=0.003 | 1.28807 | 1 |
| RANTES p<0.0005 | 0.648027 | 1 |
| IL-6 p=0.012 | 1.43189 | 1 |
| GRO-KC p=0.602 | 1.09857 | 1 |
| IL-17A p=0.240 | 1.05183 | 1 |
| MIP-1a p=0.031 | 0.698892 | 1 |
| IL-12p70 p=0.225 | 1.31263 | 1 |
| TGF-β1 p=0.225 | 1.22684 | 1 |

Overall, the manuscript is interesting and contains ample amount of data. Hence, if authors resolve the above concerns, may turn into a promising publication.

Reviewer #3: Study done by Huhtakangas et. al. entitled “A single intra-articular dose of vitamin D analog calcipotriol alleviates synovitis without adverse effects in rats”, proposed reversal of Zymosan-induced arthritis by single calcipotriol injection without obvious side effects, however, a critical revision is strongly recommended.

There is major confusion with the study time point in this study. Sometimes, author included 0h, 1h, 3h and 8h, whereas in cytokines they presented the data with only 3 time points i.e. 1h, 3h, and 8h. Moreover, Author claimed that they recorded the cytokine measurement prior to compounds treatment and presented data as day 1 data. Also, authors should need to discuss the cellular/molecular processes affected by Zymosan treatment and calcipotriol injection.

***Author response****: We thank for the comment. The reviewer obviously means the discrepancy between time points. Clinical measurements and thermal imaging were done on days 0, 1, 3 and 8 under general anesthesia. Blood was drawn only on days 1, 3 and 8 since a maximum of 10% of blood volume loss was acceptable from an individual rat.*

*The claim that cytokines were recorded prior to compounds treatment is true. Day 0 was induction of arthritis with zymosan to all 3 drug groups, and on day 1, the cytokine blood samples and drug administration (respectively) were conducted under the same general anesthesia. The reason why cytokines were not measured on day 0 was limitation in blood sampling due to volume loss.*

***Author action:*** *We agree with the final comment. The exact effects of zymosan in general are discussed in introduction, and in our experiment throughout the results section. A few lines more text have been added in Introduction to clarify the molecular mechanisms of Zymosan and vitamin D compounds.*

Any specific reason to performed under general anesthesia on days 0, 1, 3 and 8 only. It would be great if author explain why they decided to go with this timeline.

***Author response****: We thank for the comment. Days 0 and 1 were obvious choices (start of arthritis and start of drug administration), and day 3 was estimated to be convenient for finding possible clinical response on drugs. Day 8 was chosen based on a pilot study as synovial proliferation could be seen at that time-point.*

Line 34 – Author please elaborate “OA” stands for.

***Author action:*** *This is explained now.*

Intro need some editing, especially para 3.

***Author action****: We thank the notion. The nature of Zymosan arthritis is now explained better in Introduction.*

Author should include exact figure number [for example Fig 1A, Fig 1B, … Fig 2A etc].

***Author action****: The exact figure numbers are used now in the text.*

Although, the authors observed gradual weight loss in all the treated groups, why there is sudden weight loss in dexamethasone group at early stages of time-line. Author should discuss this result.

***Author action****: We thank for the comment. This is now added in Discussion. “The weight loss in general in all arthritis groups, is most probably due to loss of appetite caused by inflammation, stress and pain, and further enhanced by dexamethasone itself.”*

Table-1 – the presentation of parameter “timing” is bit confusing. Why authors recorded various parameters for the control group only at termination. 25-(OH)2D3 concentration at early phase (e.g. day 0 or day 1) is necessary.

***Author response:*** *We thank the comment. The control rats did not undergo general anesthesia until in the end since procedures like arthritis induction and drug injections were not done for controls and thus blood was only drawn during terminal anesthesia. 25-dihydroxyvitamin-D_3_ measurements were decided to be taken at termination for the same reason.*

*25-dihydroxyvitamin-D_3_ with half-life of 15 days reflects the vitamin D status and diet of the rats. All rats were eating the same food throughout the study. Therefore, 25-dihydroxyvitamin-D_3_ was measured from all rats at the end. Calcipotriol may decrease the level of 1,25-dihydroxyvitamin-D_3_ via inhibition of 1α-hydroxylase as shown in earlier studies (Knutson JC et al., Biochem Pharmacol, 1997). Therefore, the level of the biologically active hormone may transiently decline after calcipotriol dosing. 1,25-dihydroxyvitamin-D_3_ levels were not measured in this study since the chemiluminescence-based method interferes with vitamin D analog calcipotriol (hydroxyl group positions 1 are the same). The purpose of measuring of 25-dihydroxyvitamin-D_3_ levels was to verify that none of the rats had excess 25-dihydroxyvitamin-D_3_ that could potentially cause hypercalcemia.*

***Author action:*** *We removed the lowest two rows in Table 1 and added the 25-dihydroxyvitamin-D_3_ levels in the text. Now the Table 1 is easier to read.*

Fig 2, There is mismatch of figure panel sequence and figure legend.

***Author action:*** *The glucose and ca-ion panels in Figure 2 have now been exchanged.*

In Fig 2B, Why the control group has wider data distribution along Y-axis as compared to all other groups?

***Author comment:*** *We thank for the comment. The distribution of the glucose values indeed seems a bit higher in the control group. The SD of the group was 1,8; while the SD in calcipotriol-treated groups was from 1,15 to 1,72; which do not differ greatly from the control value, but the SDs in dexamethasone- and vehicle-treated groups were lower. However, considering day 1 standard deviations, we see that the SD in calcipotriol group was 1,72; while the SDs in dexamethasone and vehicle groups were 0,63 and 0,55 respectively, even though the values were taken before drug administration, i.e. the treatment was the same at the time of sampling. As the SD is between 0,55 and 1,72 in groups with similar treatment and handling, it is likely that the higher distribution of the control group is purely coincidental.*

Fig 6 – Authors showed that inflammatory cytokines were increased at day 1 in all Zymosan treated group. As Calcipotriol has antiproliferative and anti-inflammatory effects,
Authors should explain the reason behind the increased expression of both pro- and anti-inflammatory cytokines on day one. Moreover, Authors did not explain on what basis they selected the cytokines to test against the treatment. A table with complete statistical values is required.

***Author action:*** *We thank for the comment. The cytokine figure was redesigned to show the differences between arthritic and nonarthritic rats (as the day 1 measurements were taken prior to drug dosing). The statistical values are now shown. The cytokines had increased expression on day 1 as arthritis was induced on day 0.*

***Author response:*** *The levels of both pro- and anti-inflammatory cytokines are induced by Zymosan on day 1 before drug administration (Fig 6). The net effect of all mediators determines the biological response. The immune system is able to quench the acute arthritis after 1-2 weeks by shifting the balance from proinflammatory to anti-inflammatory direction. A sentence about this is now added in Discussion: “Anti-inflammatory cytokines like IL-13 and IL-4 that were also induced by Zymosan may contribute to tissue repair and quenching of arthritis within 1-2 weeks.”*

*The cytokine panel was the same as in the previous experiment of the research group, and it was considered to be useful in quantifying relevant cytokines induced by synovial cells upon inflammation. The whole cytokine analysis is now presented in a clearer manner concerning arthritic and nonarthritic rats, rather than concentrations between groups, in which there were few differences.*

Authors reported that VEGF level significantly reduced in calcipotriol group, however, error bars in the graph (Fig 6) is not going along with the statement.

***Author action:*** *We thank for the comment. The VEGF level was calculated with Kruskal-Wallis test between arthritis groups (p=0.046). The pairwise comparisons were nonsignificant, and this is now explained in the text. “There were no statistically significant differences in the changes in cytokine levels across the arthritis groups, except for VEGF level which declined the most in the calcipotriol group during days 1–8 (p=0.046), but this difference was not significant in pairwise comparisons.”*

Authors should present a dose dependent result that supports their selection of Zymosan dosing of 0.1 mg.

***Author action:*** We thank for the comment. This is now explained in Methods as follows:*“Initially, a dose of 0.5mg was administered in the pilot study to two rats, but the arthritis was considered clinically too severe for the purposes of the experiment and considering the 3Rs principle.”*

Authors has presented format table of clinical scoring but it is not clear that how the scoring distributed. Marely presenting the format sheet does not make any sense. Additionally, Fig S2 need better presentation. X- and Y-axis are missing and strongly needed to make figure understandable.

***Author action:*** *We thank for the comment. The distribution of scores is shown in figure S2. We have presented the figure in a clearer manner and written a reference to figure S2 in the legend of the scoring sheet and vice versa. The average clinical score in all arthritic groups remained <1 throughout the experiment, therefore the score in figure S2 is presented as a sum over the whole experiment. This is now indicated in the legend. Titles for x- and y-axes have been added.*

*New legends:*

***S1 Table. Clinical scoring sheet of the rats, English translation.*** *The scoring sheet was used for clinical scoring on a daily basis for each arthritic rat. The results are shown in S2 Fig.*

***S2 Fig. The clinical scores of the rats during the study.*** *A.* *The sum of clinical scores on each day during the experiment in different arthritis groups. Comparing the nine-day-sum of all scores, there was no significant difference between the arthritis groups (p=0.881 in Kruskal-Wallis test). B. The amount of rats according to the nine-day sum score at the end of the experiment. All of the rats had relatively low scores and showed practically no pain behaviour or difficulties in moving. The scores were given daily to each arthritic rat using the scoring sheet in S1 Table. The average scores were <1 every day in every group and thus, the scoring is represented as sums.*
